# Supplementary material for: How useful is a complete urinary tract ultrasound in orchiepididymitis?
Source: PLoS One. 2022 Feb 10;17(2):e0263934. doi: 10.1371/journal.pone.0263934 (PMC8830649; doi:10.1371/journal.pone.0263934)
Supplement: Attachment — Submitted filename: Response to Reviewers.docx [file pone.0263934.s001.docx]

**How useful is a complete urinary tract ultrasound in orchiepididymitis?**

**Response to reviewers**

Dear Editor and Reviewers,

Thank you for your feedback, suggestions and questions. Please find below our answers to the different points you raised.

Regards,

Dr Eva Aeschimann on behalf of the research team

*Reviewer #1: The submitted article presents useful data for clinical practice. The submitted statistical analysis is valid, showing diagnosis of GU tract anomalies to be rare. I do not routinely obtain upper tract imaging in patients presenting with orchitis, but there is debate about this, especially in younger boys. The submitted article adds to the evidence that upper tract imaging is not needed.*

Dear Reviewer #1, thank you for your positive feedback. We hope too that the submitted article will contribute to taking forward discussion on OE management.

*Reviewer #2: I found this to be a well written and clinically useful manuscript. In addition to the screening urinary tract US data, other findings in this patient population included a very low incidence of UTI (3.1%), inflammatory markers that were indeterminate and noncontributory, and 75% of patients had improved symptoms after 48 hours. In trying to understand this improvement, the role of treatment received from the ER in terms of antibiotic and pain management is unclear. Was there a consistent ER protocol for treatment? Were antibiotics given only when the urinalysis was positive? Was there a standard pain management regimen? The 3 documented UTIs occurred in infants less than 6 months of age. Although they had negative urinary tract ultrasounds, would you advise further evaluation with a VCUG in these patients after the first OE episode?

Minor corrections: The study dates given in the Abstract are 2011-2017 while in the Methods and Results the dates are 2012-2017. Also, in figure 1 the dates are 2011-2017.*

Dear Reviewer #2, thank you for your positive feedback and questions.

Antibiotic and pain management: In our pediatric center, all children diagnosed with OE receive a prescription of paracetamol. Regarding antibiotic treatment, it is limited to urinary tract infections (positive urinanalysis). We have added this information in the manuscript.

VCUG: In our center we follow the Swiss consensus recommendations on urinary tract infections in children. VCUG is only conducted when an anomaly is detected by a renal and urinary tract ultrasound or in case of recurrent UTI infections. We would not advise further evaluation with a VCUG after the first OE episode.

Minor corrections: the correct time frame is 2012-2017. We have corrected this information in the abstract and in figure 1.
